# Supplementary material for: Bioconvergence of sound-guided and supramolecular assembly strategies to create peptide-protein composite hydrogels with predictable shape-to-function features
Source: Mater Today Bio. 2025 Dec 9;36:102643. doi: 10.1016/j.mtbio.2025.102643 (PMC12813217; doi:10.1016/j.mtbio.2025.102643)
Supplement: Multimedia component 1 [file mmc1.docx]

Supplementary Material

**Bioconvergence of sound-guided and supramolecular assembly strategies to create peptide-protein composite hydrogels with predictable shape-to-function features**

Cosimo Ligorio^1,2,3,4^, Alessandro Cianciosi^5^, Riccardo Tognato^5^, Micaela Natta^5^, Romedi Parolini^5^, Sena Ardicli^6,7^, Huseyn Babayev^6^, Ieva Sapjanskaite^2^, Samantha L. Kilgour^8^, Richard Homer^9^, Bapan Pramanik^2^, Zhiyu Zhou^10,11^, Andrea Malandrino^12^, Eleni Priglinger^12^, Cezmi Akdis^6^, Martin J. Stoddart^5^, Alvaro Mata*^1,2,3,4^ and Tiziano Serra*^5,13^

**Affiliations:**

^1^Biodiscovery Institute, University of Nottingham, Nottingham, UK

^2^School of Pharmacy, University of Nottingham, Nottingham, UK

^3^Department of Chemical and Environmental Engineering, University of Nottingham, Nottingham, UK

^4^NIHR Nottingham Biomedical Research Centre, Nottingham, NG7 2UH UK

^5^AO Research Institute Davos, Switzerland

^6^Swiss Institute of Allergy and Asthma Research (SIAF), University of Zurich, Davos, Switzerland

^7^Department of Genetics, Faculty of Veterinary Medicine, Bursa Uludag University, Bursa, Turkey

^8^School of Chemistry, University of Nottingham, Nottingham, UK

^9^Wolfson Building, University of Nottingham, Nottingham, UK

^10^Innovation Platform of Regeneration and Repair of Spinal Cord and Nerve Injury, Department of Orthopaedic Surgery, The Seventh Affiliated Hospital, Sun Yat-sen University, Shenzhen, China;

^11^Guangdong Provincial Key Laboratory of Orthopaedics and Traumatology, Orthopaedic Research Institute/Department of Spinal Surgery, The First Affiliated Hospital of Sun Yat-sen University, Guangzhou, China

^12^Johannes Kepler University Linz, Kepler University Hospital, Department of Orthopaedics and Traumatology, Linz, Austria

^13^Complex Tissue Regeneration Department, MERLN Institute for Technology-Inspired Regenerative Medicine, Maastricht University, Maastricht, Netherlands

***Corresponding Author:**

**Alvaro Mata**

Biodiscovery Institute, School of Pharmacy and Department of Chemical and Environmental Engineering, University of Nottingham, Nottingham, UK

Email: [a.mata@nottingham.ac.uk](mailto:a.mata@nottingham.ac.uk)

**Tiziano Serra**

AO Research Institute Davos, Switzerland and Complex Tissue Regeneration Department, MERLN Institute for Technology-Inspired Regenerative Medicine, Maastricht University

Email: [tiziano.serra@aofoundation.org](mailto:tiziano.serra@aofoundation.org)

**Figures**

**
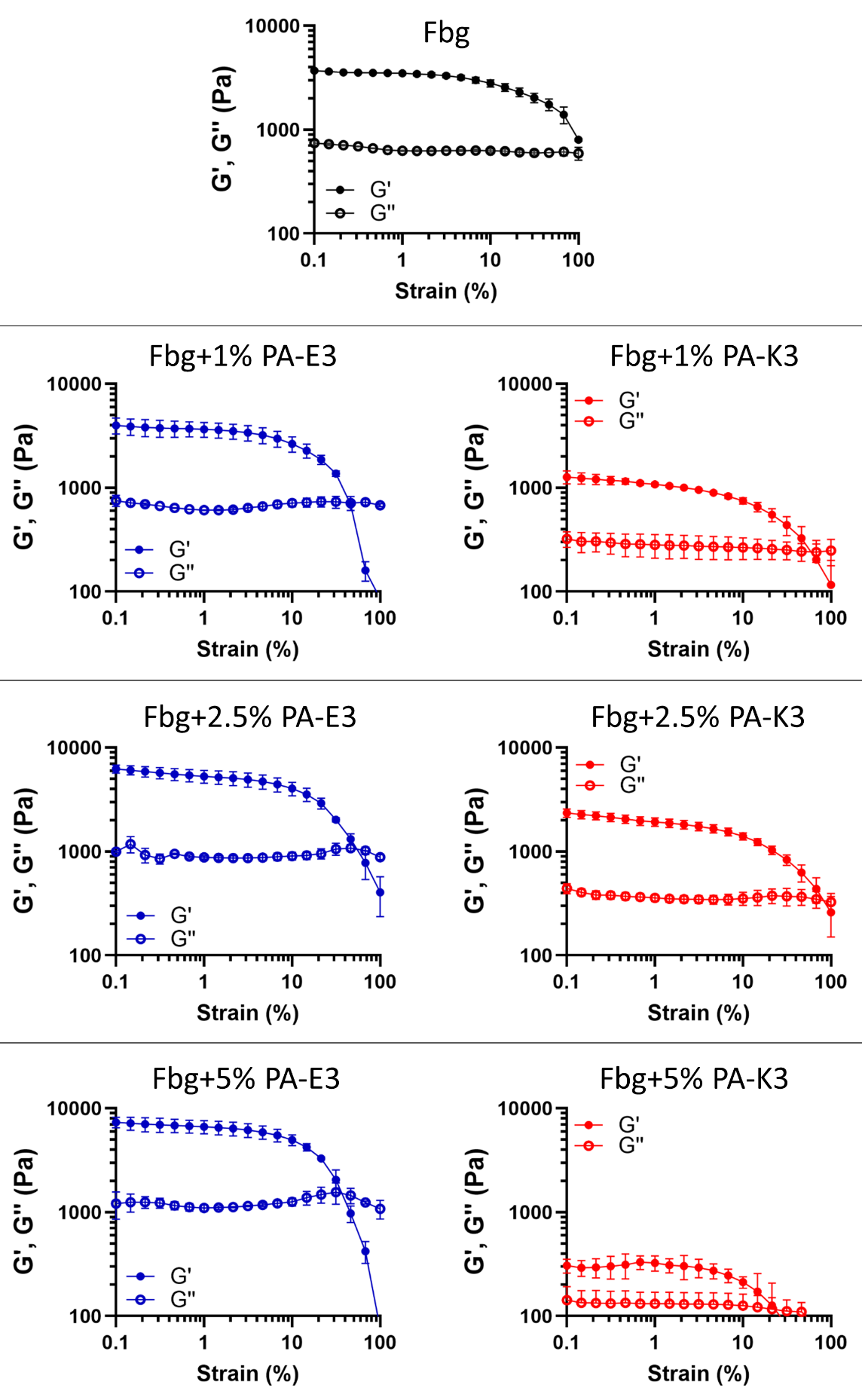
**

***Figure*** ***S1. Strain sweep tests of PA-fibrin gels.*** *(Top) Oscillatory rheology tests of strain sweeps were performed for fibrin (top, black curves), PA-E3-fibrin gels (left side, blue curves) and PA-K3-fibrin gels (right side, red curves).*

**
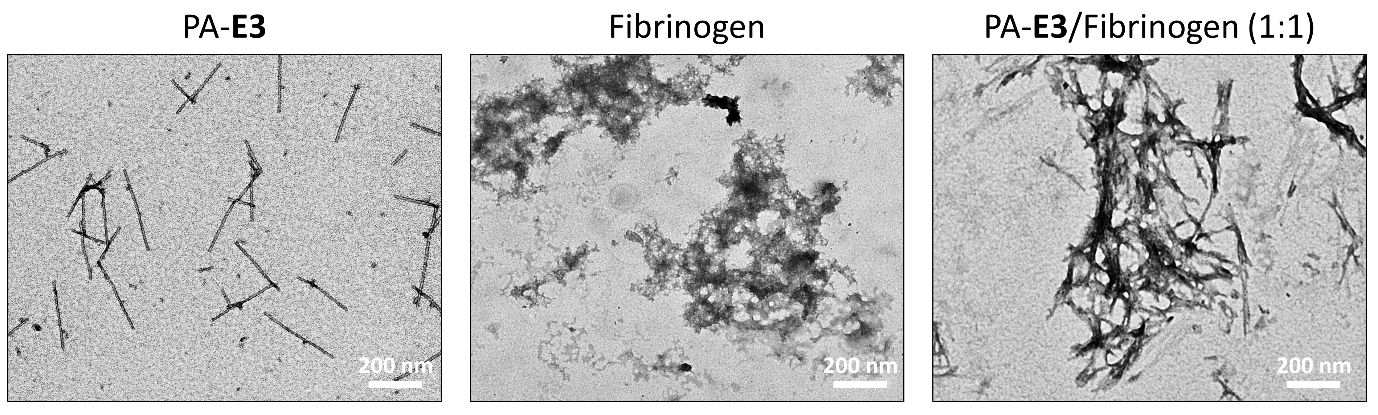
**

***Figure*** ***S2.*** ***TEM micrographs for PA-E3, fibrinogen and PA-E3/fibrinogen mixture.*** *Images show that mixing PA-E3 and fibrinogen before thrombin and CaCl_2_ addition leads to the formation of co-assembled complexes, which provide a structural basis for the accelerated gelation observed at increasing PA-E3 concentrations.*


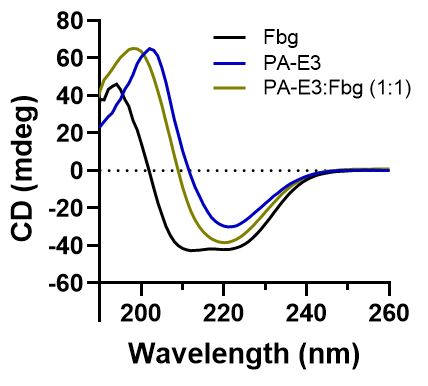


***Figure*** ***S3.*** ***Circular dichroism of PA, fibrinogen and PA-fibrinogen mixture.*** *CD spectra of PA-E3 (blue) show* *characteristic β-sheet conformation, while fibrinogen (black) displays an α-helix secondary structure. Upon mixing, PA-E3 and fibrinogen undergo significant re-arrangement of their secondary structure (gold spectrum).*


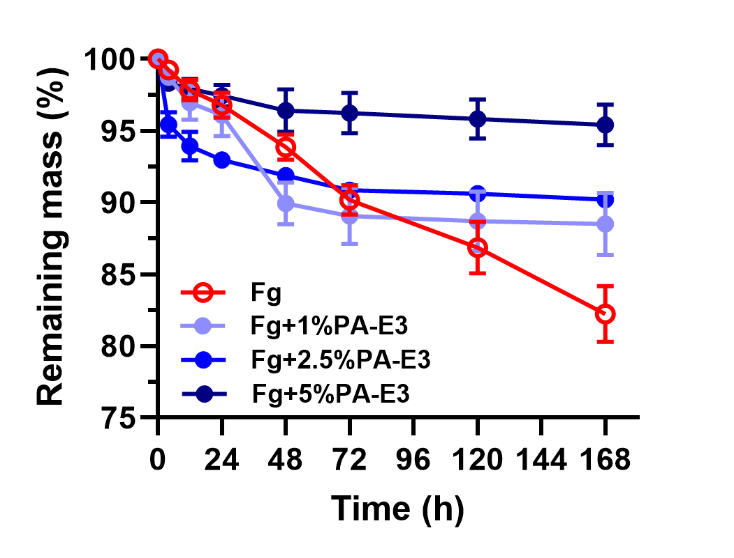


***Figure*** ***S4.*** ***Enzymatic hydrogel degradation.*** *Degradation over time of fibrin (Fg) and hybrid fibrin gels obtained by mixing fibrinogen with increasing concentrations of PA-E3 before addition of thrombin.*


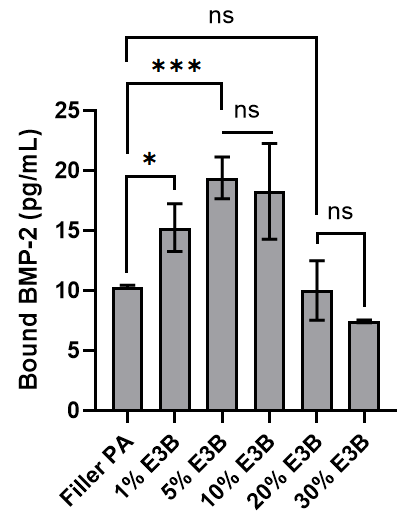


***Figure*** ***S5. BMP binding capability of PA-E3B nanofibres.***  *ELISA tests of PA-E3B-BMP-2 solutions shows bound BMP-2 and the capability of PA-E3B nanofibres to bind free BMP-2 under different epitope densities. Data presented as mean ± SD, n=3, compared with unpaired t test, n=3, ^ns^p > 0.05, *p < 0.05, **p < 0.01, ***p < 0.001.*

*
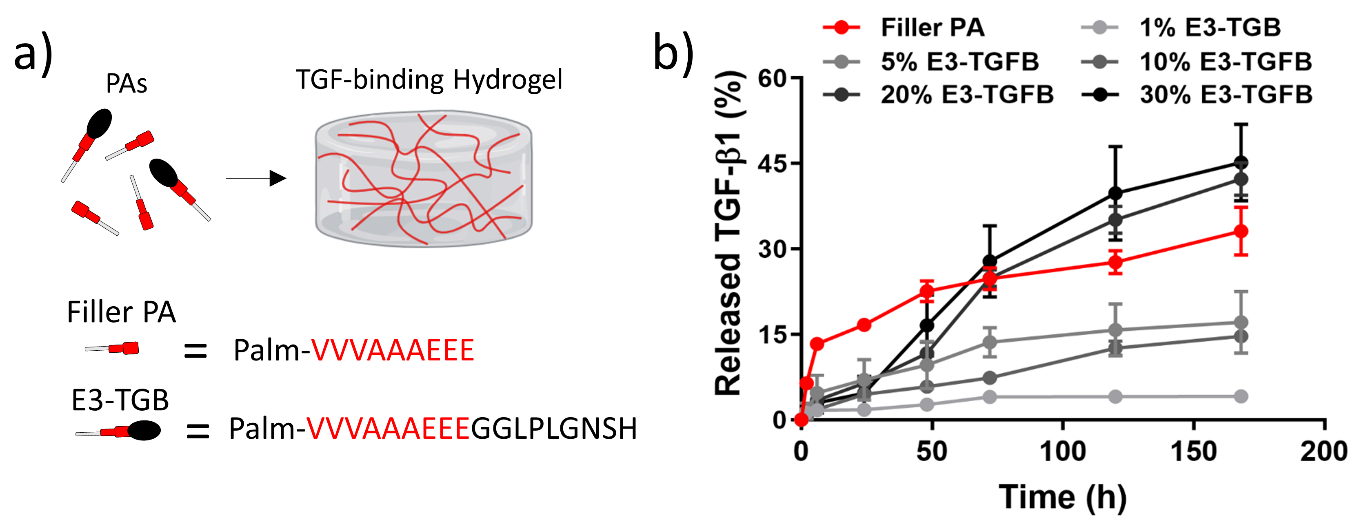
*

***Figure*** ***S6.*** ***Release of bound TGFβ1****. a) PA sequences used to bind and release TGFβ1. b) ELISA assays of TGFβ1 released from 3D hydrogels containing PA-E3 only (‘filler PA’) and gels containing increasing weight ratios of TGFβ1-binding peptides (‘E3-TGB’, i.e. PA-E3 displaying the GF-binding peptide sequence ‘**LPLGNSH’). Graphs show dependence of release profiles on epitope density.*

*
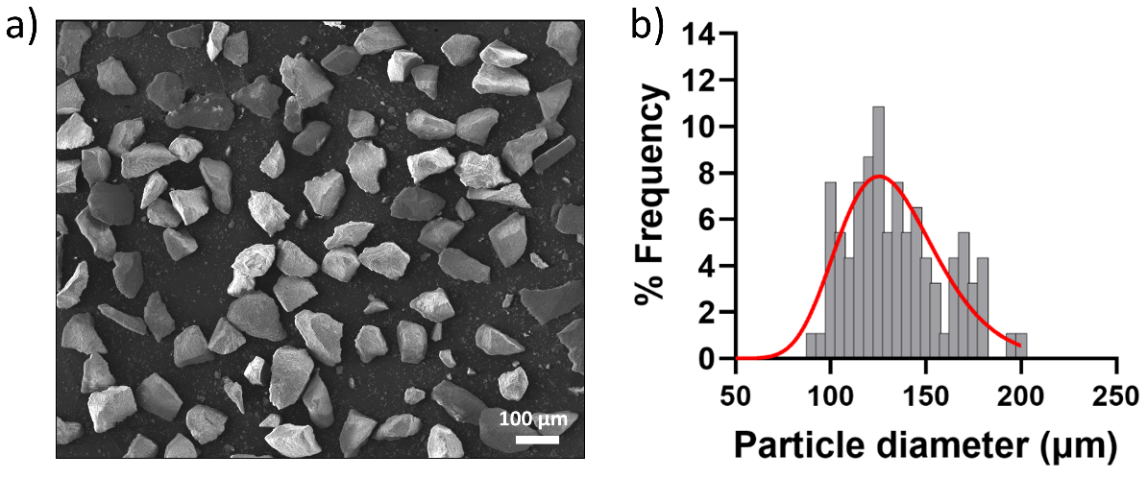
*

***Figure*** ***S7.*** ***Size distribution of calcined bone particles (CBPs)****. a) SEM image of CBPs on a carbon tape. b) Frequency distribution of the CBP size shows the majority of particles lying between 80 and 180 µm in size.*


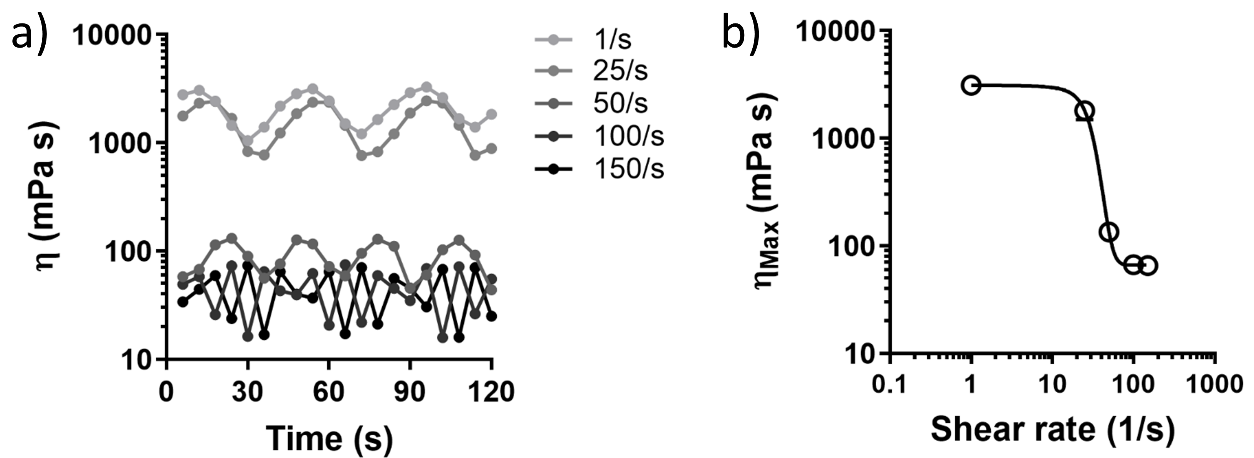


***Figure*** ***S8.*** ***Rheological behavior of PA-fibrinogen solution****. a) Flow sweep measurements of PA-fibrinogen solution recording viscosity () while varying time and frequency of test oscillations. b) Maximum viscosity extracted from the maxima of graph a) in function of shear rates. Both graphs shows time-dependent and shear-thinning behaviour of the PA-fibrinogen solution used.*


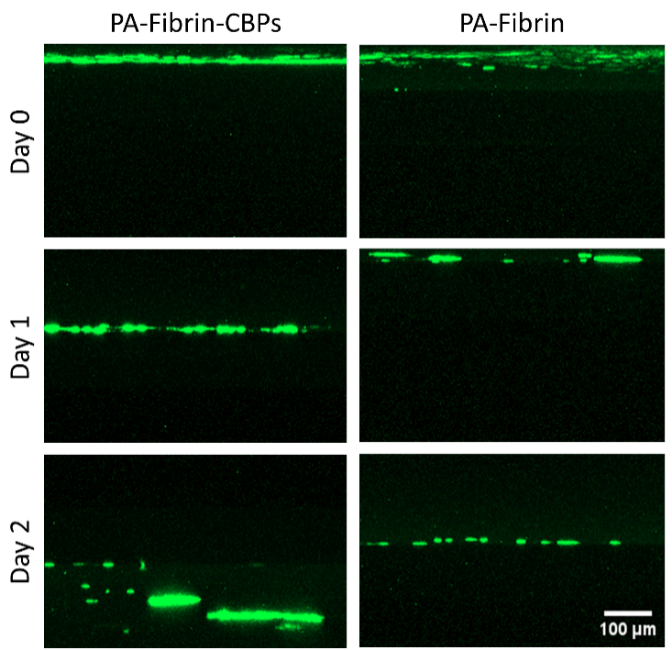


***Figure*** ***S9. Migration of hMSCs within the hydrogels****. Images shows the migration over time of fluorescent hMSCs, which have been stained with calcein AM and seeded onto the surface of PA-Fibrin-CBPs and PA-Fibrin.*

**Tables**

**Table S1.** List of frequency and peak-to-peak voltage values used to obtain the proposed patterns.

| **Frequency (Hz)** | **Peak-to-peak voltage (V)** |
| --- | --- |
| 25 | 0.454 |
| 57 | 0.0874 |
| 75 | 0.0505 |
| 79 | 0.0459 |
| 92 | 0.0336 |
| 101 | 0.0279 |
| 143 | 0.0139 |

**Videos**

***Video S1.*** *Numerical simulations of the liquid surface displacement induced by Faraday waves.*

***Video S2.*** *Real-time video of CBP pattern generation within a solution of PA-fibrinogen.*

***Video S3.*** *Stretching of PA-fibrin-CBP hydrogel membranes using a pair of tweezers. Samples could be stretched multiple times without any visible pattern loss and detachment of the embedded CBPs.*

***Video S4.*** *FEM simulations of patterned membranes at 0 Hz showing Von Mises stress maps****.***

***Video S5.*** *FEM simulations of patterned membranes at 101 Hz showing Von Mises stress maps****.***

***Video S6.*** *FEM simulations of patterned membranes at 143 Hz showing Von Mises stress maps****.***

***Video S7.*** *Real-time video of MSC spheroids pattern generation within a solution of PA-fibrinogen.*
